# Wisconsin Society of Veterinary Graduates

**Published:** 1896-01

**Authors:** J. P. Laws, W. G. Clark

**Affiliations:** President, 309 E. Main St., Madison, Wis.; Secretary, Beaver Dam, Wis.


Secretary.

### WISCONSIN SOCIETY OF VETERINARY GRADUATES.

THE annual meeting will be held in Madison, Wednesday, February 5, 1896, at 1.30 P.M. Programme: "Shoeing," Dr. E. L. Morgenroth; "Scientific Breeding," J. R. Kelso; "Equine Relapsing Fever," T. W. Watson; "Veterinary Meat-inspection by the State and its Necessity," G. Ed. Leech; "Professional Ethics," J. P. Laws.

The meeting of the State Agricultural Society will be held the same week and excursion rates will be given by the railroad companies.

You are cordially invited to be present at this meeting.

J. P. LAWS, President,
309 E. Main St., Madison, Wis.

W. G. CLARK, Secretary,
Beaver Dam, Wis.

AMONG other attractions at the annual meeting of the Michigan State Association, on February 4th, at Lansing, will be to have practical demonstrations of the "Operation for Roaring," "Spaying," "Castration of Cryptorchids," etc.

